# Altered spinogenesis in iPSC-derived cortical neurons from patients with autism carrying *de novo SHANK3* mutations

**DOI:** 10.1038/s41598-018-36993-x

**Published:** 2019-01-14

**Authors:** Laura Gouder, Aline Vitrac, Hany Goubran-Botros, Anne Danckaert, Jean-Yves Tinevez, Gwenaëlle André-Leroux, Ekaterina Atanasova, Nathalie Lemière, Anne Biton, Claire S. Leblond, Aurélie Poulet, Anne Boland, Jean-François Deleuze, Alexandra Benchoua, Richard Delorme, Thomas Bourgeron, Isabelle Cloëz-Tayarani

**Affiliations:** 1 Human Genetics and Cognitive Functions, Institut Pasteur, Paris, France; 2 CNRS UMR 3571 « Genes, Synapses and Cognition », Institut Pasteur, Paris, France; 3 Université Paris Diderot, Sorbonne Paris Cité, Human Genetics and Cognitive Functions, Paris, France; 4 Imagopole, Citech, Institut Pasteur, Paris, France; 5 MaIAGE, INRA, Université Paris-Saclay, 78350 Jouy-en-Josas, France; 6 Bioinformatics and Biostatistics Hub, C3BI, USR 3756 IP CNRS, Institut Pasteur, Paris, France; 7 CECS, I-STEM, AFM, 91030 Evry Cedex, France; 8 Centre National de Recherche en Génomique Humaine (CNRGH), Institut de Biologie François Jacob, CEA, Université Paris-Saclay, F-91057 Evry, France; 9 Assistance Publique-Hôpitaux de Paris, Robert Debré Hospital, Department of Child and Adolescent Psychiatry, Paris, France

## Abstract

The synaptic protein SHANK3 encodes a multidomain scaffold protein expressed at the postsynaptic density of neuronal excitatory synapses. We previously identified *de novo SHANK3* mutations in patients with autism spectrum disorders (ASD) and showed that *SHANK3* represents one of the major genes for ASD. Here, we analyzed the pyramidal cortical neurons derived from induced pluripotent stem cells from four patients with ASD carrying *SHANK3 de novo* truncating mutations. At 40–45 days after the differentiation of neural stem cells, dendritic spines from pyramidal neurons presented variable morphologies: filopodia, thin, stubby and muschroom, as measured in 3D using GFP labeling and immunofluorescence. As compared to three controls, we observed a significant decrease in *SHANK3* mRNA levels (less than 50% of controls) in correlation with a significant reduction in dendritic spine densities and whole spine and spine head volumes. These results, obtained through the analysis of *de novo SHANK3* mutations in the patients’ genomic background, provide further support for the presence of synaptic abnormalities in a subset of patients with ASD.

## Introduction

Autism spectrum disorders (ASD) are characterized by atypical social communications, and the presence of restricted and repetitive patterns of behavior. ASD frequently co-occur with additional comorbidities such as intellectual disability (ID) and epilepsy^1^. The genetic architecture of ASD differs among individuals, ranging from apparently monogenic to polygenic forms^2^. Among the genes recurrently found mutated in individuals with ASD, the *SHANK* genes code for synaptic scaffolding proteins located at glutamatergic synapses. *SHANK* mutations can occur in patients with ASD with an intelligence quotient in the normal range (*SHANK1*), but mostly in patients with mild (*SHANK2*) to severe *(SHANK3)* ID^3^. *SHANK3* haploinsufficiency also contributes to the clinical symptoms of patients with Phelan-McDermid syndrome (PMS) characterized by a terminal deletion of chromosome 22q13 that includes *SHANK3* in the large majority of cases^4^. *SHANK3* mutations represent a relatively frequent genetic cause for ASD accounting for 1–2% of individuals with ASD and ID^3,5^. In addition, truncating *SHANK3* mutations were also reported in rare cases of schizophrenia^6^.

SHANK proteins are located at postsynaptic densities (PSD) of excitatory neurons in different brain areas^7^. They are cytoskeletal scaffold proteins involved in the formation/function of dendrites. SHANK3 recruits a large number of synaptic intracellular interactors such as Homer as well as several neurotransmitter receptors including the α-amino-3-hydroxyl-5-methyl-4-isoxazole-propionic acid (AMPA), the metabotropic glutamate (mGlu), and the *N*-methyl-D-aspartic acid (NMDA) glutamate receptors^8^. On average, each single glutamatergic synapse contains approximately 300 SHANK family proteins (SHANK1-3), which correspond to approximately 5% of the total number of synaptic proteins^9^. A defect in the expression of one SHANK member causes significant synaptic deficiencies as evaluated by using animal models and cultured neurons. In mice, disruption of the *Shank3* gene alters excitatory synaptic transmission and leads to autistic-like behaviors such as social communication deficits and repetitive behaviors^10–14^. By contrast, overexpression of Shank3 in cultured aspiny cerebellar neurons triggers the development and maturation of functional spines expressing NMDA, AMPA and metabotropic glutamatergic receptors^15^. This overexpression approach in rat neurons in culture was also used to characterize the functional impact of SHANK mutations on the reduction of dendritic spines density^16,17^. In a recent study, Mei *et al*.^18^ generated a conditional knock-in mouse model in which the re-expression of the *Shank3* gene restores spine densities in the adult striatum. In this model, the social communication deficits and the repetitive grooming behaviors were also specifically restored. By contrast, motor coordination and anxiety remain unchanged. All together, both *in vitro* and *in vivo* data indicate a significant role of SHANK3 protein in the regulation of synaptic plasticity with possible reversion of the observed deficits at the adult stage.

In humans, six studies investigated the role of SHANK3 using induced pluripotent stem cells (iPSC). Results from four studies in which iPSC-derived neurons from patients were used showed a reduction in dendritic spine or excitatory synaptic transmission as compared with controls^19–22^. These effects can be reversed by SHANK3 overexpression, insulin growth factor 1 (IGF1) treatment^19^, Cdc2-like kinase 2 (CLK2) inhibition^20^ or lithium treatment^21^. Another study using iPSC-derived enterocytes revealed a decrease in zinc uptake transporter that could play a role in the gastro-intestinal symptoms of the patients^23^. It should be noted that most of these studies used cells from patients with PMS who were carriers of relatively large 22q13.3 deletions, encompassing not only *SHANK3* but also additionnal genes that could also contribute to the phenotype^4^. One study used genome-editing technologies to introduce *SHANK3* single nucleotide mutations in iPSC-derived neurons from controls^24^. The authors showed that heterozygous and homozygous *SHANK3* truncating mutations severely impair hyperpolarization-active cation channels (HCN) that are associated with reduced synaptic connectivity and transmission^24^. All these results converge towards a synaptic deficit in patients with ASD carrying a *SHANK3* mutation, but none of the previous studies has investigated the dendrite morphology of neurons carrying *SHANK3* truncating mutations in the patient genetic background. In the present study, we selected four independent patients carrying heterozygous truncating *de novo SHANK3* point mutations who had initially been characterized in our laboratory^3^. We generated the corresponding iPSCs for their selective reprogramming into cortical neurons in order to examine the effects of *SHANK3* haploinsufficiency on the level of *SHANK3* mRNA and on the 3D spine morphogenesis organization.

## Results

### Characterization of iPSC-derived neurons from patients with *SHANK3 de novo* mutations

**Figure 1:**
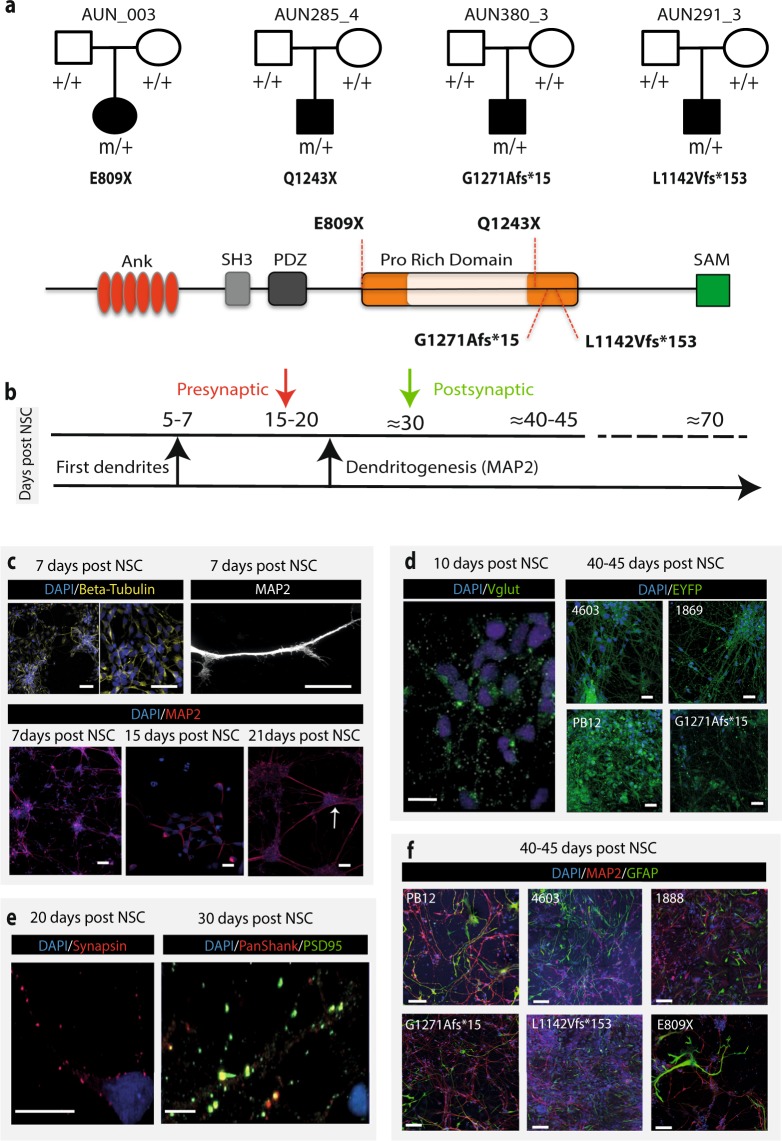
Pedigrees of the families carrying *de novo* SHANK3 mutations and neuronal characterization. (**a**) Upper, the four patients probands carry *de novo* truncating mutations in *SHANK3* gene (two « STOP » and two « frameshift » mutations leading to a premature STOP codon). (**a**) Lower, A schematic representation of the multidomain SHANK3 protein with the location of the four mutated aminoacids is provided. Conserved domains are indicated by filled rectangles. The mutations are located within the Proline-rich structure of SHANK3, between the PDZ and the SAM domains, and within exon 21 of *SHANK3* gene. (**b**) Schematic representation of neuron maturation at different time periods. (**c**) Upper left, Immunofluorescence staining of human control iPSC cells-derived neurons by beta III tubulin at 7 days post NSC differentiation. (**c**) Upper right, labeling of a single dendrite using the MAP2 marker showing dendritic growth cones at early stages of culture. (**c**) Lower, Immunofluorescence staining of human iPSC cells-derived neurons by MAP2 at different intervals of time post NSC differentiation. The target shows neurite elongation and established connectivity between cell clusters. (**d**) VGlut1 marker at 10 days post NSC differentiation stained glutamatergic neurons.  Labeling of control (1869, 4603, PB12) and ASD (G1271Afs*15) mature neurons at 40–45 days post NSC differentiation using the pAAV-CaMKIIa-hChR2-EYFP-WPRE lentivirus are also illustrated. (**e**) Immunofluorescence staining using presynaptic (synapsin) and postsynaptic (PSP95 and PanSHANK) markers. No staining was detectable before the indicated days post NSC differentiation (20 days for Synapsin, 10 days for VGlut1 and 30 days for PSD95). Data are from at least two control individuals (PB12 and 4603) (**f**) Immunofluorescence GFAP staining of cultures from iPSC-derived neurons showing the presence of astrocytes in 3 controls individuals (PB12, 4603, 1888) and 3 patients (G1271Afs*15, L1142Vfs*153, E809X). Scale bars: 10 μm (a-e); 100 μm (f).

We used the human iPSC-based model to analyze the effects of heterozygous truncating *de novo SHANK3* mutations found in four patients with ASD, and presenting moderate to severe ID. Their clinical symptoms are fully described in Leblond *et al*.^3^. The schematic location of the selected mutations in SHANK3 protein is shown in Fig. 1a. The four mutations are predicted to lead to truncated proteins either by inducing a stop codon directly (E809X and Q1243X) or via a frameshift of the open reading frame (G1271Afs*15 and L1142Vfs*153). For clarity, the six ankyrin domains are presented in their 3D structure to illustrate precisely their number and complete juxtaposition (Supplementary Fig. 1). Indeed, some contradictory results have been published regarding the topological characteristics of the ANK domains^25,26^. Other SHANK3 domains such as SH3, PDZ and SAM are also complex structures that are involved in the interaction of SHANK3 proteins with its intracellular partners^26^. Supplementary Fig. 2 illustrates the main technical steps for fibroblasts reprogramming into pluripotent stem cells, commitment to the dorsal telencephalon lineage, derivation, amplification and banking of late cortical progenitors (LCP) as previously described^27^. LCP were differentiated into cortical neurons of the superficial layers II-IV, which correspond to the cortical upper layers as confirmed previously by Cux1, Cux2 and Brn2 immunolabeling^27^. We used fibroblasts from patients with E809X and Q1243X mutations^27^ and fibroblasts from one patient with G1271Afs*15 mutation^21^. The characterization of iPSC derived from the fibroblast of patient L1142Vfs*153 was not reported previously and is presented in Supplementary Fig. 3. In addition, we derived similar cortical glutamatergic neurons from three independent control individuals, namely 1869, 4603 and PB12^21,27^. Supplementary Fig. 2 illustrates the different steps of culture that allow the production of a significant percentage of mature neurons and study workflow. Figure 1b shows the different maturation steps of the neurons obtained at 40–45 days post neural stem cells (NSC) differentiation. We first checked for the absence of any significant variability in the growth of neurites between single culture samples using the MAP2 staining^28,29^ (Fig. 1c and Supplementary Fig. 4). According to our protocol, iPSC were predominantly derived into glutamatergic neurons as illustrated by VGlut1 immunoflorescence staining and the selective expression of the yellow fluorescent protein drived by the calcium/calmodulin-dependent protein kinase II (CaMKII) promoter (Fig. 1d). This promoter is predicted selective for pyramidal excitatory neurons *vs*. inhibitory interneurons^30^. In accordance with Boissart *et al*.^28^, these neurons accounted for 70–80% of total cells whereas GABAergic neurons accounted for about 15% (data not shown). Our data clearly indicate a predominant presence of glutamatergic cells in the culture, independently of the cellular phenotypes (CTR *vs*. ASD). Finally, we determined the appearence of presynaptic and postsynaptic components by synapsin and PSD95 immunofluorescence staining. Colocalisation of SHANK and the excitatory marker PSD95 was first detectable at 30 days after NSC differentiation (Fig. 1e). We observed that neuronal cells could be kept in culture for about 70 days after NSC differentiation. However, they were not always at a full cellular viability. The time slot of 40–45 days therefore appeared convenient to study dendritic spine maturation and morphometry while preventing any cellular death. At this stage of maturation, we could also detect the presence of astrocytes accounting about 5–10% of total cells in control and ASD neuronal cultures (Fig. 1f). Astrocytes are known for their role in promoting the maturation of NSC^31^. They influence the local environment of iPSC-derived neurons, their maturation and promote synaptogenesis^32^.

### *SHANK3* expression in iPSC-derived neurons from patients and control individuals

**Figure 2:**
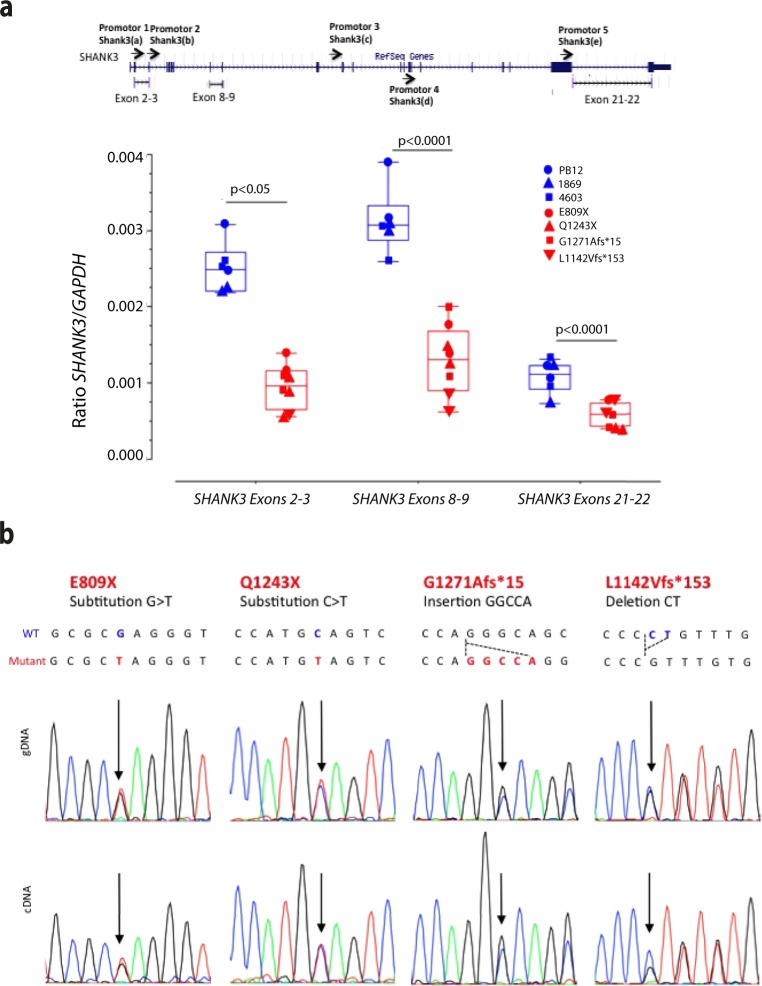
Analysis of *SHANK3* gene expression in iPSC-derived neurons from controls and ASD patients. (**a**) Quantification of *SHANK3* mRNA in iPSC-derived neurons (40 days post NSC) using RT-ddPCR. Data are mean ± SEM. Statistical analysis was performed using a two-way Anova. *p < 0.05 ***p < 0.001 (**b**) Sequencing diagrams showing the presence of the four mutations in the genomic and cDNA extracted from iPSC-derived neurons.

We investigated *SHANK3* expression in iPSC-derived neurons from control individuals and patients. *SHANK3* mRNA were quantified using droplet digital PCR (ddPCR)^33^ after reverse transcription (RT) of mRNA from cells in culture. *SHANK3* mRNA levels were significantly decreased by an average of 25% to 50% in patients as compared with controls (p < 0.001) (Fig. 2). RNAseq experiments show similar decrease in *SHANK3* mRNA levels as well as an enrichment of under-expressed genes among the genes targeted by Fragile-X mental retardation protein (FMRP) (Supplementary Fig. 5). By contrast, housekeeping genes were not enriched in genes which were differently expressed in neurons from ASD patients as compared with control neurons (Supplementary Fig. 5). We then sequenced the genomic and cDNA to detect the level of *SHANK3* mutated allele (primers are presented in Supplementary Table S1). Surprisingly, the mutant allele was present with a similar ratio between mutant and wild-type alleles in all patients’ cDNA (Fig. 2). These data also indicate that under our experimental conditions, the nonsense-mediated mRNA decay (NMD) mechanism devoted to the elimination of mRNA transcripts with premature stop codons appears inefficient. This might reflect previous observations of NMD dysfunction during neuronal cell development^34^. Studies would be necessary to confirm the decrease in the levels of SHANK3 proteins in samples from patients.

### Spinogenesis in pyramidal neurons derived from iPSC is correlated to SHANK3 expression

**Figure 3:**
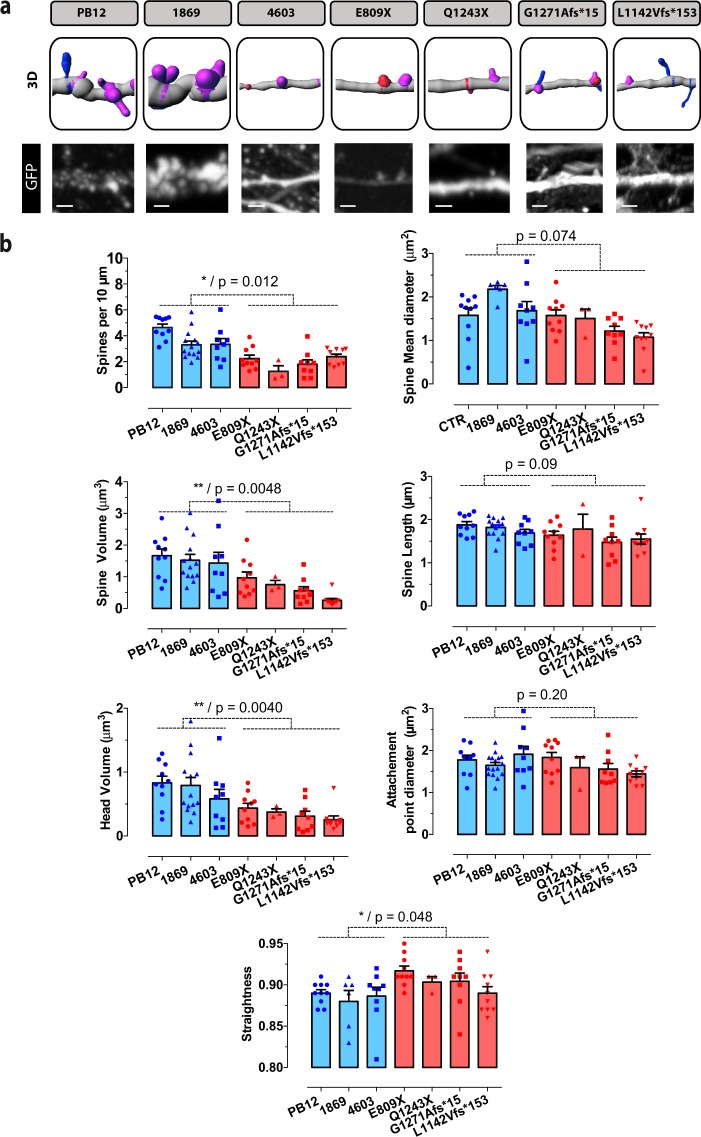
Quantitative analysis of the morphological parameters of primary dendritic spines between control and ASD neurons. (**a**) Primary spine segments with corresponding 3D reconstructions. Scale bar = 2 μm. Dendrite segments (grey color) are endowed with four categories of spines: Filopodia (pink color), Thin (blue), Stubby (pink) and Muschroom (green). (**b**) Spine morphological parameters were quantified using the Imaris sofware as described in Materials and Methods. Numbers of neuronal dendrites are indicated in the graph. Data are presented as mean ± SEM. Statistical analysis was performed using unpaired Student t-test in order to analyze the significance between mean values from combined controls and combined patients. Equality of variances was checked using the Fisher’s F-test. P values are directly indicated in the graph. *p < 0.05, **p < 0.01.

**Figure 4:**
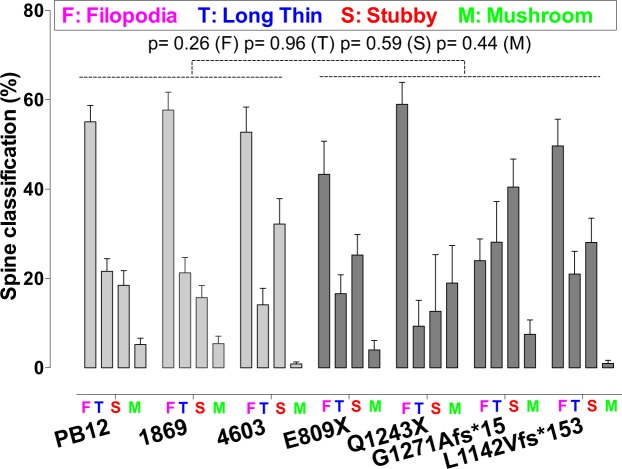
Morphological classification of primary dendritic spines between control and ASD neurons. Morphological parameters of the four spine categories were quantified using the Imaris sofware as described in Materials and Methods. Data are presented as mean ± SEM. Statistical analysis was performed using unpaired Student t-test. Equality of variances was checked using the Fisher’s F-test. P values are directly indicated in the graph. Combined mean values for Filopodia were significantly increased as compared to combined values from all other spine categories (p < 0.0001). Statistical analysis was performed as described above.

**Figure 5:**
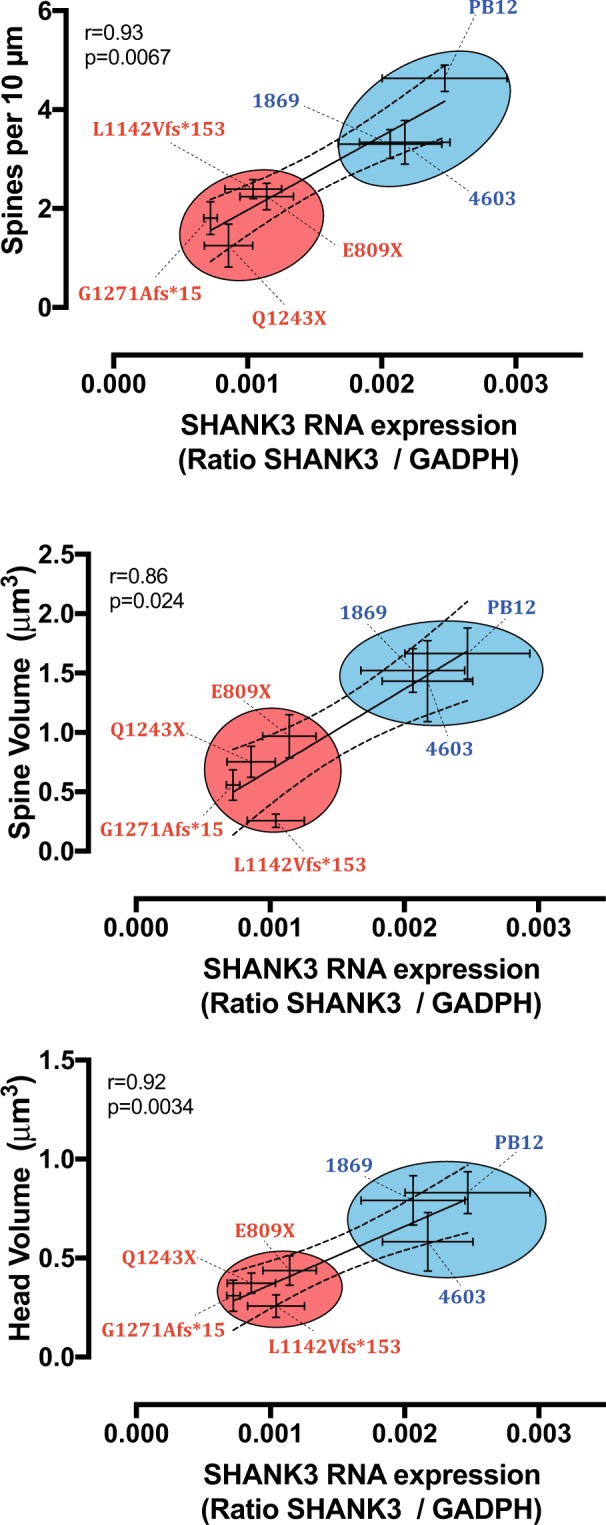
Correlation analysis between SHANK3 expression and alterations in dendritic spine parameters in iPSC-derived neurons from control individuals and patients with ASD. Upper graph, Correlation between *SHANK3* mRNA and spine densities. Middle graph, Correlation between SHANK3 mRNA and total spine volume. Lower graph, Correlation between *SHANK3* mRNA and head spine volume. Statistical analysis was performed using GraphPad Prism Version 6 software (GraphPad, sand Diego, California, USA). Data are mean ± SEM. Coefficients were calculated using Spearman correlation method and are indicated in the graphs with statistical significance.

Reduced SHANK3 levels are expected to modify dendritic spine morphology. We therefore measured spinogenesis in iPSC-derived pyramidal glutamatergic cells by comparing the primary dendrites of neurons from patients with ASD to those from three control individuals (Fig. 3a,b). Our data show a significant decrease of nearly 50% in spine densities in all the four patients as compared to the three control individuals. The total volume of spines, as well as their head volume was also decreased to a similar extent. Spine straightness was slightly increased in patients but this change was less pronounced than those observed on spine densities and volumes. Spine mean diameter and spine length as well as the surface of spine attachment to the dendrite remained globally constant between patients and control individuals (Fig. 3b). Our data indicate that all spine categories were represented in pyramidal neurons from both patients and controls. However, we observed some differences in their relative proportions: filopodia were present at highest densities in both control individuals and patients as compared to the combined thin, stubby and muschroom categories (p < 0.0001). By contrast, we did not observe any significant difference in spine maturation between patients and controls (Fig. 4). When *SHANK3* mRNA levels and dendrites were analyzed together, we found a significant correlation between the level of *SHANK3* mRNA and spine density (p = 0.0067), spine volume (p = 0.024) and spine head volume (p = 0.0034) (Fig. 5).

## Discussion

In brain cortical circuits, dendritic spines play an important role in the establishement of excitatory synapses. However, their quantitative morphological analysis has been poorly documented in iPSC-derived neurons from patients with ASD. We therefore analyzed spinogenesis in iPSC-derived pyramidal glutamatergic cells using our published method for the 3D quantification of GFP-labeled dendritic spines^35^. So far, data from studies using iPSC-derived neurons in neurodevelopmental disorders have included only one or two patients. In our study, we have increased the number to four ASD patients with precise *SHANK3* mutations. This aspect is of importance since each individual has a unique genetic background. In addition, a broad range of severe clinical symptoms are observed in patients carrying *SHANK3* mutations^3,4,36^. Finally, recent observations indicate a dysregulation of different microbial species in Shank3 KO mice^37^ that may affect GABAergic system^38^ together with an apparent complex relationship between *SHANK3* genotype, sex and immune response^37^. These observations strengthen the importance of using a larger number of patients for the analysis of cellular phenotypes in humans.

We could observe altered 3D spine morphologies and densities in comparison with control neurons in iPSC-derived neurons from four patients with ASD carrying *SHANK3 de novo* truncating mutations. Moreover, we could also correlate the reduced expression of *SHANK3* mRNA in neurons reprogrammed from patients’fibroblasts with the effects of the four distinct *SHANK3* mutations on spinogenesis. In neuronal cells, a dendritic mRNA transport occurs with local translation at dendritic spines^39^. The decrease in *SHANK3* mRNA levels may result from reduced spine densities as observed in reprogrammed neurons from patients. The spatio-temporal control of gene expression which occurs at synapses involves the FMRP protein. FMRP target genes which are also subject to dendritic mRNA transport with local translation at synaptic sites^40^ were enriched in down-regulated genes.

Our data are in agreement with the findings of Shcheglovitov *et al*.^19^ showing fewer excitatory synapses in iPSC-derived neurons obtained from two patients with PMS carrying a complete deletion of *SHANK3* as compared to controls. Our data are also in agreement with those reported in rat neurons by Durand *et al*.^16^ who demonstrated by a 2D spine analysis, that a stop *SHANK3* mutation selectively reduced the density of spines along the dendrites, while spine width remained unchanged. In the study of Bidinosti *et al*.^20^ using rat cortical neurons, spine densities were in the same of order as those from our study. Moreover, these authors found that inactivating *SHANK3* led to a similar reduction in spine densities. Altered dynamics of maturation of spines from excitatory neurons during development may underlie the cortical dysfunctions found in ASD by modifying the intracortical projections of excitatory neurons. We could observe that spine maturation is not fully achieved. Filopodia were present at the highest densities in controls and in patients, as compared to other spine categories. The distribution of spine categories in patient G1271Afs*15 was slightly different with apparent lower levels of filopodia as compared with other individuals. Dendritic spine dysgenis in neurons from ASD patients may underlie the synaptic defects found in diverse mouse models of ASD^41,42^. An inadequate organization of neuronal circuits may cause the main clinical behavioral deficits, also reproducible in mouse models. At molecular levels, the genetic manipulation of selected proteins such as Shank3 but also Shank2, as well as neurexins, neuroligins, Epac2, Tsc1/2, Ube3A, and PTEN that are involved in ASD, leads to altered spine shapes and densities in rodent models^43^. Current advances in SHANK3 protein research clearly correlate its deficit to a wide-range of human neurological disorders with developmental synaptic dysfunction that may also be associated with synaptic decline at later stages as observed in Alzheimer disease^44^. The fact that Shank3 protein has been shown to differentially regulate brain cortical and striatal circuits in mice^45^, further supports its complex involvement not only in ASD but also in other neurological disorders.

In conclusion, our results obtained on iPSC-derived neurons from four independent patients with ASD and ID fully support the deleterious role of *SHANK3* mutations on dendrite density and morphology in the patient genetic background. This abnormality of dendrites is a current feature, which is also observed in iPSC-derived neurons from patients with neurodevelopmental syndromes (i.e. PMS, Rett syndrome, Timothy syndrome, Fragile X syndrome) as reviewed elsewhere^46–49^. The 3D measurement of the dendritic spines morphologies represents a useful index for synaptic defects in patients with neurodevelopmental disorders.

## Methods

We confirm that all methods were carried out in accordance with relevant guidelines and regulations. We confirm that all experimental protocols were approved by the named institutions. Informed consent was obtained from all subjects as detailed below.

### Ancestry analysis of patients

For the ancestry analysis, patients and control individuals were genotyped using Illumina Infinium Omni1/2.5 (1 M/2.5 M SNPs) and Illumina Infinium Humancore24 (300 K SNPs) beadchips, respectively. To assess the genetic background of patients and controls, genotyping data from HapMap3 populations was used as a reference panel and the genetic distance based on pairwise identity by state calculation was performed using PLINK and 17 K SNPs (overlapping SNPs from the Illumina technologies) (Supplementary Fig. 6).

### Production of human pluripotent Stem cells and their derivation into cortical neurons of layer II to IV

The iPSC were produced as described previously^27^. Following the patient’s legal representatives’ approval, 8-mm skin punch biopsies were obtained (study approval by Committee for the Protection of Persons, CPP no. C07-33). The clinical characteristics have been published^3^. Fibroblasts reprogramming was performed using the four genes coding for the human factors *OCT4*, *SOX2*, *c-Myc*, and *KLF4* cloned in Sendai viruses (Invitrogen). Induced PSC lines were characterized according to Boissart *et al*.^27^. Commitment of pluripotent stem cells (PSC) to the neural lineage and derivation of stable cortical neural stem cells (NSC) was also described previously^27,35^. For the control iPSC lines, we used GM04603 and GM01869, two male fibroblast cultures obtained from the Coriell Biorepository (Coriell Institute for Medical Research, Camden, NJ, USA). The control line PB12 was reprogramed from peripheral blood mononuclear cells obtained from an anonymous female blood donor at the French Blood Donor Organization.

### Immunofluorescence

Immunofluorescence was described in Gouder *et al*.^32^. The list of primary and secondary antibodies with their respective dilutions is presented in Supplementary Table S2.

### Lentiviral transduction

The protocol for the transduction of PGK-GFP-lentivirus has been previously described^32^. The same experimental conditions were used for the pAAV-CaMKIIa-hChR2-EYFP-WPRE lentivirus without subsequent immunofluorescence against the yellow fluorescent protein. The CaMKIIa promoter is predicted to drive specific expression in neurons *vs*. glia and in pyramidal excitatory neurons *vs*. inhibitory interneurons^28^. Transduction of neuronal cultures was performed using a lentivirus containing a calcium/calmodulin-dependent protein kinase II (CaMKII) promoter provided by Dr Gabriel Lepousez.

### Primary dendritic spine imaging

For imaging of dendritic spines, a step-by-step protocol has been previously published and is available in a video journal^32^. Main steps are as follows: (1) transduction of iPSC-derived cells with GFP lentiviral vectors followed by an immunofluorescence labeling with an anti-GFP antibody; (2) confocal imaging by a confocal laser-scanning microscope using a 40X oil NA = 1.3 objective and a 488 nm laser for GFP excitation; (3) selection of neurons with a pyramidal morphology; (4) Z-stack acquisitions with a Z spacing ranging from 150 nm to 300 nm; (5) Semi-automatic tracing of dendrites and automated segmentation of spines using the Filament Tracer module of Imaris 7.6 software (Bitplane AG, Zürich); (6) Spine classification in four classes defined by their morphology as follows: Stubby: length < 1μm; Muschroom: Length (spine) > 3 μm and Max width (head) > mean width (neck) x 2; Long thin: Mean width (head) ≥ Mean width (neck); Filopodia-like: length > 1 μm (no head).

### Genomic, cDNA sequencing, and Genotyping

For genomic DNA sequencing, cells were lyzed in lysis buffer (50 mM Tris, pH = 8.0, 20 mM NaCl, 1 mM EDTA, 1% SDS) with proteinase K. DNA was extracted using phenol-chloroform isoamylalcool (ref. P2069, Sigma) and isopropanol (ref. 59309-1L, Sigma). For cDNA sequencing, RNA was extracted using the miRNA Micro Kit (QIAGEN) and reverse transcription was performed using the SuperScript ® VILO cDNA Synthesis Kit (ref. 11754–250, Invitrogen). The region encompassing the mutation was amplified by PCR using 5 or 4 sets of primers for the genomic DNA and cDNA, respectively (Supplementary Table S1) and the Kapa2G polymerase (KAPA 2G Robust HotStart PCR Kits, ref. KK5517, Clinisciences). PCR products were run on agarose gels and purified using phosphatase (FastAP, ref. EF0651, ThermoScientific) and exonuclease enzymes (Exonuclease I, EN0581, ThermoScientific). Controls were performed in the absence of reverse transcriptase. Sequencing was performed using the BigDye®TerminatorV3.1 cycle sequencing and purifications kits (ref. 4337455,4376484, ThermoFisher).

### RT-ddPCR assay

RNAs from 40-day human neurons in culture were extracted using the miRNA Micro Kit (QIAGEN, ref. 217004) and converted in cDNA using the Superscript ® VILO cDNA synthesis Kit (ref. 11753–250, Invitrogen). The expression of *SHANK3* mRNA was analyzed using 3 sets of primers and probes targeting respectively exons 2-3, 8-9 and 21-22. A duplex ddPCR strategy was used to amplify and quantify *SHANK3* and the housekeeping gene *GADPH* in a same well. For each set of primers targeting *SHANK3*, probes were coupled either with the FAM dye or with the HEX dye. Each reaction medium contained 10 μl ddPCR Supermix for probes (without UTP), 1 μl of *SHANK3* primer/probe mix (Biorad), 1 μl of GADPH assay (Biorad), 6 μl of nuclease free water, and 2 μl of DNA (2.5 ng/μl). 20 μl of each reaction medium was transferred to a droplet generation cartridge before adding 70 μl of Droplet Generation Oil. Droplets generated with QX200 Droplet Generator were loaded into a clean 96-well PCR plate, sealed with foil. The PCR reaction conditions were as follows: 1 cycle at 95 °C for 10 min, 40 cycles at 94 °C for 30 s, 1 cycle at 55 °C for 1 min, followed by 1 cycle at 98 °C for 10 min. Droplet signals were obtained using a Bio-Rad QX200 droplet digital PCR system (Bio-Rad, USA). Data were analyzed using the Bio-Rad QuantaSoft software version 1.3.2. Negative controls were obtained by excluding the retrotranscription step. Up to 20000 discrete droplets were analyzed for each PCR sample (Supplementary Table S3).

### Statistics

The sample size and statistical analyses are reported in each figure legend. 95% confidence levels have been used.

## Supplementary information

Supplementary Information

## Data Availability

The data that support the findings of this study are available from the corresponding author on reasonable request.
