# Supplementary material for: Altered spinogenesis in iPSC-derived cortical neurons from patients with autism carrying de novo SHANK3 mutations
Source: Sci Rep. 2019 Jan 14;9:94. doi: 10.1038/s41598-018-36993-x (PMC6331634; doi:10.1038/s41598-018-36993-x)
Supplement: Supplementary file 1 — Supplementary Information [file 41598_2018_36993_MOESM1_ESM.pdf]

Altered spinogenesis in iPSC-derived cortical neurons from patients with autism carrying *de novo SHANK3* mutations

Laura Gouder[1,2,3$], Aline Vitrac[1,2,3$], Hany Goubran-Botros[1,2,3$], Anne Danckaert[4], Jean-Yves Tinevez[4], Gwenaëlle André-Leroux[5], Ekaterina Atanasova[1,2,3], Nathalie Lemière[1,2,3], Anne Biton[6], Claire S. Leblond[1,2,3], Aurélie Poulet[7], Anne Boland[8], Jean-François Deleuze[8], Alexandra Benchoua[7], Richard Delorme[1,9], Thomas Bourgeron[1,2,3] and Isabelle Cloëz-Tayarani[1,2,3*]

[1]Human Genetics and Cognitive Functions, Institut Pasteur, Paris, France
[2]CNRS UMR 3571 « Genes, Synapses and Cognition », Institut Pasteur, Paris, France
[3]Université Paris Diderot, Sorbonne Paris Cité, Human Genetics and Cognitive Functions, Paris, France
[4]Imagopole, Citech, Institut Pasteur, Paris, France
[5]MaIAGE, INRA, Université Paris-Saclay, 78350, Jouy-en-Josas, France
[6]Bioinformatics and Biostatistics Hub, C3BI, USR 3756 IP CNRS, Institut Pasteur, Paris, France
[7]CECS, I-STEM, AFM, 91030 Evry Cedex, France
[8]Centre National de Recherche en Génomique Humaine (CNRGH), Institut de Biologie François Jacob, CEA, Université Paris-Saclay, F-91057, Evry, France
[9]Assistance Publique-Hôpitaux de Paris, Robert Debré Hospital, Department of Child and Adolescent Psychiatry, Paris, France

[$]These authors contributed equally to the manuscript

Figure S1: 3D structure of selective domains of SHANK3 protein

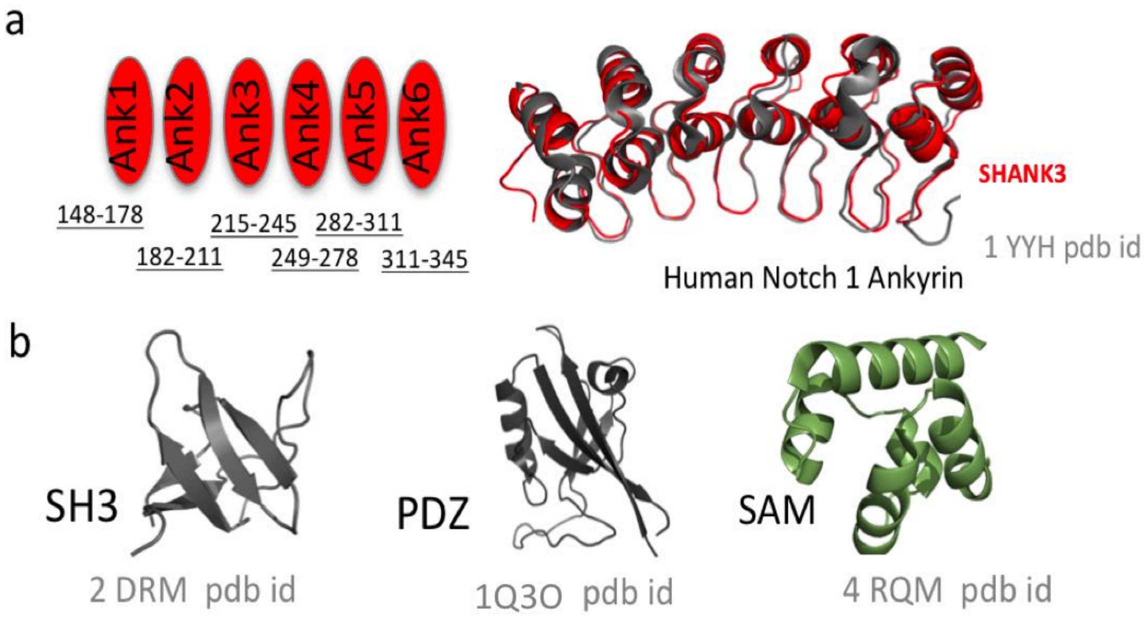

Legend to Supplementary Figure S1: a. Left panel shows the delineation for the six ankyrin (ANK) domains ranging from residues 148 to 345 within the SHANK protein aminoacid structure. Right panel shows the juxtaposed ANK domains, which have been homology modeled according to a method reported previously[28,29], using 1YYH (Ehebauer et al, 2005), 3B7B (Collins et al, 2004) and 1WDY (Tanaka et al, 2004) pdb identities as 3D templates, selected from HHPred detection (Söding et al, 2005). **b.** Illustration of the 3D structures of the SH3, PDZ and SAM domains, which are identified domains of full-length SHANK. All FASTA sequences were retrieved from the UnitProtKB server at http//www.uniprot.org/uniprot/Q9BYBO#structure.

Figure comment: The six ankyrin (ANK) domains are presented in their putative 3D structure to illustrate precisely their numbers and complete juxtaposition with respect to ANK topology. Indeed, some contradictory results have been published regarding the topological characteristics of the ANK domains (Schuetz et al, 2004; Mameza et al, 2013). Although predicted benign, coding-sequence variants within the ANK domains have been identified in patients with ASD[3]. Other SHANK3 domains such as SH3, PDZ and SAM are also complex structures that are involved in the interaction of SHANK3 proteins with its intracellular partners (Mameza et al, 2013).

Supplementary References:

Collins, R.E., Northrop, J.P., Horton, J.R., Lee, D.Y., Zhang, X., Stallcup, M.R. & Cheng, X. The ankyrin repeats of G9a and GLP histone methyltransferases are mono- and dimethyllysine binding modules. *Nat. Struct. Mol. Biol* **15**, 245-250 (2004).

Ehebauer, M.T., Chirgadze, D.Y., Hayward, P., Martinez-Arias, A. & Blundell, T.L. High-resolution crystal structure of the human Notch1 ankyrin domain. *Biochem J.* **392**, 13-20 (2005).

Mameza, M.G., Dvoretskova, E., Bamann, M., Honck, H.H., Guler, T., Boeckers, T.M., Schoen, M., Verpelli, C., Sala, C., Barsukov, I. *et al*. SHANK3 gene mutations associated with autism facilitate ligand binding to the Shank3 ankyrin repeat region. *J. Biol. Chem*. **288**, 26697-26708 (2013).

Schuetz, G; Rosario, M., Grimm, J., Boeckers, T. M., Gundelfinger, E.D. & Birchmeier, W. The neuronal scaffold protein Shank3 mediates signaling and biological function of the receptor tyrosine kinase Ret in epithelial cells. *J. Cell Biol*.**167,** 945-952 (2004).

Söding, J., Biegert, A., Lupas, A. N. The HHpred interactive server for protein homology detection and structure prediction. *Nucleic Acids Res*. 33 (Web Server issue), W244–W248 (2005).

Tanaka, N., Nakanishi, M., Goto, Y., Kitade, Y. & Nakamura, K.T. Structural basis for recognition of 2’5’-linked oligoadenylates by human ribonuclease L. *EMBO J.*, **23**, 3929-39-38 (2004).

Figure S2: Study workflow

a

Production of cortical pyramidal neurons from a single clone

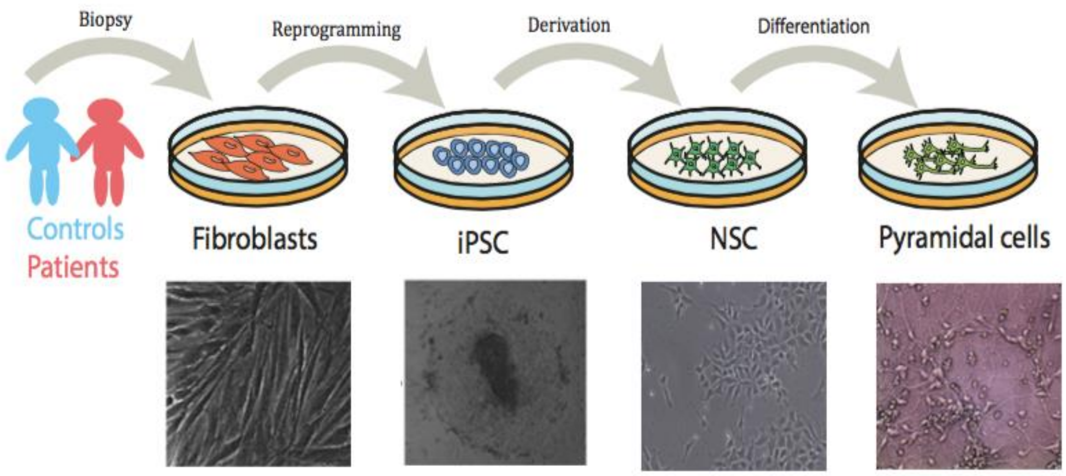

b

Analysis

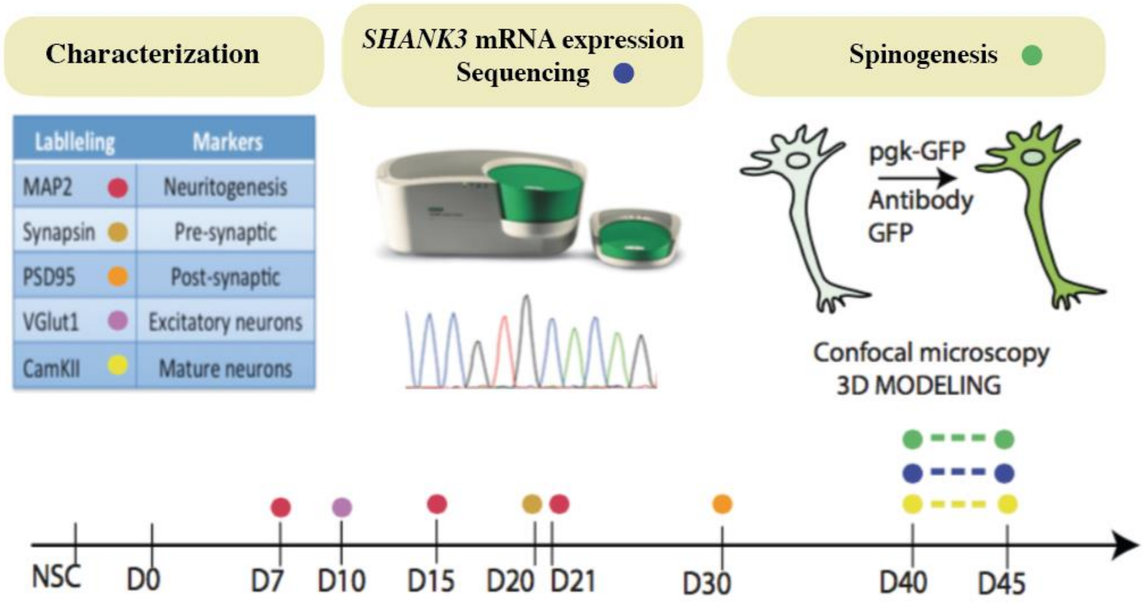

Legend to Supplementary Figure S2: a. Schematic representation of the different steps for the production of pyramidal neurons. A single clone was analyzed for each independent individual: 3 controls and 4 patients (7 cell lines in total). **b**. Illustration of the different steps of analysis, which includes neuronal characterization, *SHANK3* mRNA expression, and morphometric analysis of dendritic spines.

Figure S3: Characterization of iPSC from patient L1142Vfs*153

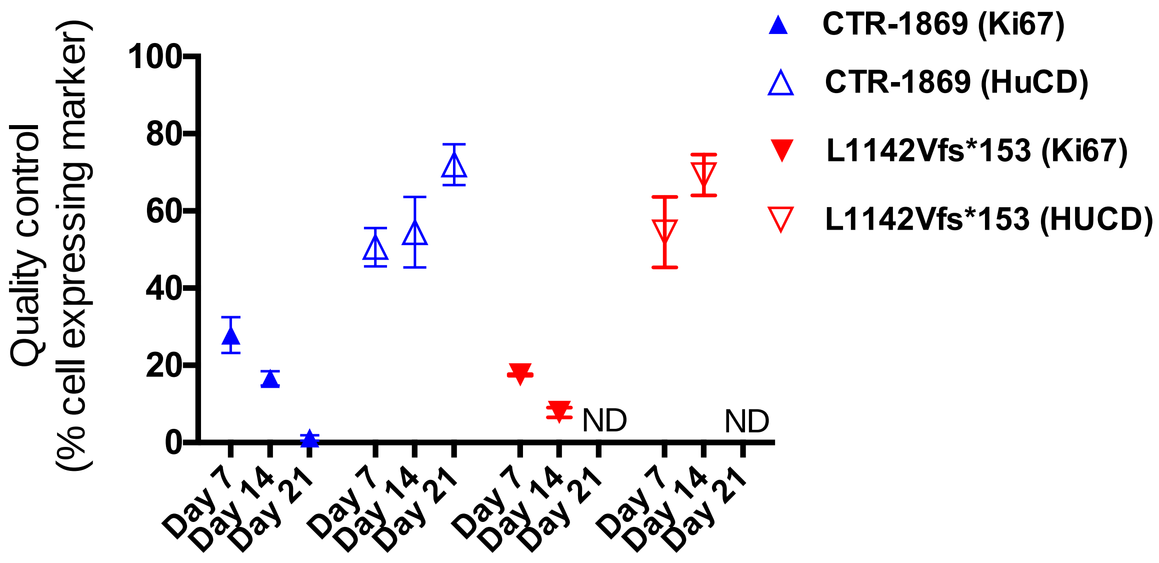

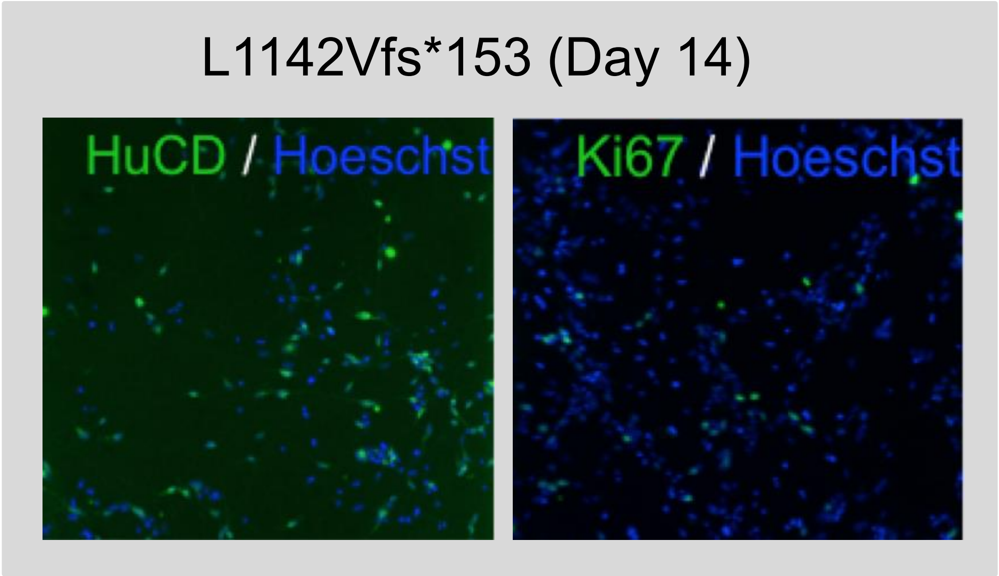

Legend to Supplementary Figure S3: Late cortical progenitors (LCP) terminal differentiation using high-throuput screening in 384-well plates as described in Boissart et al[27]. Top, Values from the graph correspond to the automated quantification of the kinetics of neural proliferation (Ki-67 marker) and neuronal differentiation (HuCD marker) at different days after cell plating for one control and patient with the *SHANK3* mutation L1142Vfs*153. Results represent the percentage of cells expressing each marker in total cell populations. Data are mean ± SD. Bottom, Representative pictures captured by the Arrayscan microscope are presented for the cells from patient L1142Vfs*153 which was not included and validated elsewhere[21,27].

Figure S4: Quantitative analysis of neuritogenesis in five distinct cultures of control human iPSC-derived neurons

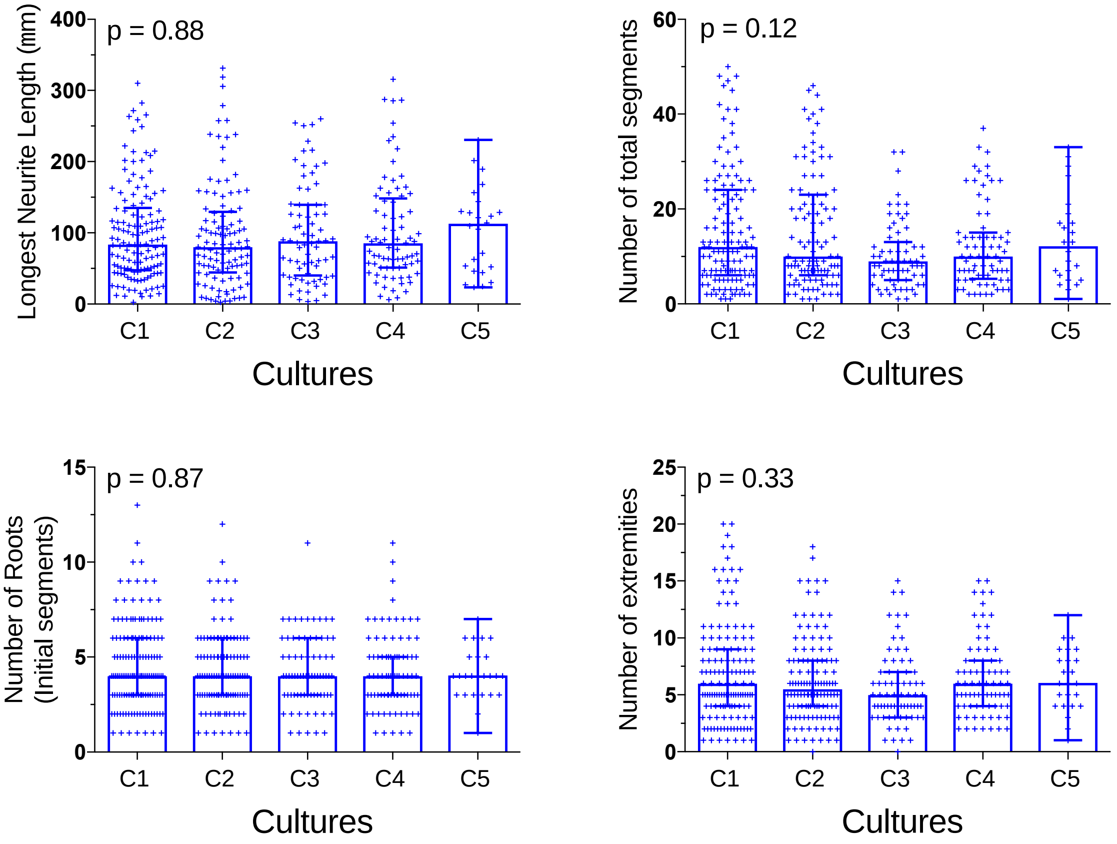

Legend to Supplementary Figure S4: Neuritogenesis, a complex dynamic process combining neurite outgrowth and branching, was followed in 5 distinct cultures of control iPSC-derived neurons (15 days post NSC). At the end of culture, neurons were fixed and immunostained with an anti-MAP2 antibody. Fluorescence images were acquired with an inverted Axio Observer.ZI (Carl Zeiss, Le Pecq, France) equipped with an AxioCam camera. Quantification of images was performed using the Acapella software (Perkin Elmer) as previously described[28]. Briefly, neuronal nuclei were detected according to the DAPI staining and for quantification all parameters were measured under the same defined threshold conditions. Measured parameters loaded from the specific module “neurite detection” include the length of the longest neurite per neuron, the total numbers of root extremities and segments. Scatter dot plots with a line at median with interquartile range are represented. Each dot represents one neuron. Statistical analysis was performed using a Kruskal-Wallis test and GraphPad Prism Version 6 software (GraphPad, sand Diego, California, USA). Outliers were identified using the ROUT method with a Q value of 1%.
P values are directly indicated in the graphs. No statistical difference was found between the 5 cultures (C1 to C5).

Figure S5: *SHANK3* gene expression in iPSC-derived neurons from controls and ASD patients.

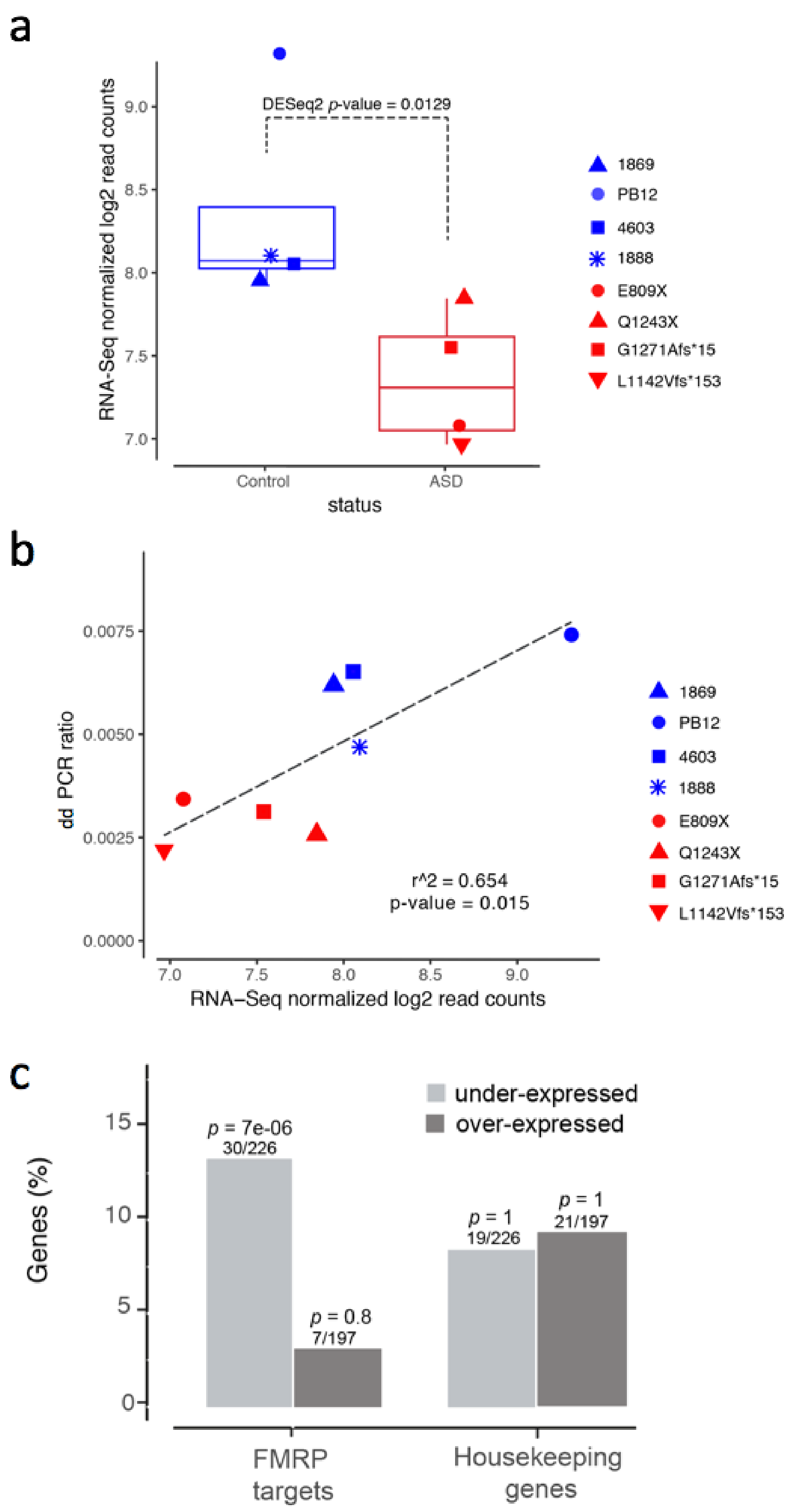

Legend to Supplementary Figure S5: **a.** Quantification of *SHANK3* mRNA in iPSC-derived neurons (40 days post NSC) using RNAseq technology. Data are shown as log2 read counts normalized by library size. Statistical analysis was performed using DESeq2 package (v1.18.1), p= 0.013. **b.** Scatter plots showing *SHANK3* expression as measured by ddPCR (Ratio *SHANK3/GAPDH*) versus RNA-Seq (log2 normalized read counts). PCR were done in two replicates for each individual. For each replicate, *SHANK3*/*GAPDH* ratio was summed across the selected *SHANK3* exons (2-3; 8-9; 21-22) to obtain one estimate of *SHANK3* expression per sample. *SHANK3* expression values were then averaged across the two PCR replicates from a same individual. Coefficients of linear regression is r2= 0.654, p=0.015. **c**. Enrichment analysis within the following two gene sets: 777 FMRP targets from Darnell et al. (2001) and 3519 housekeeping genes from Eisenberg et al. (2013). Analysis was performed on the genes within the two gene sets which were detected using DESeq2 package and observed as under-expressed (FDR < 5% and logFC < 0) and over-expressed (FDR < 5% and logFC > 0) between control and ASD neurons. A total of 15,665 genes was detected as expressed in the RNA-Seq data using a threshold of one count-per-million reads in at least three samples. The y-axis gives the percentage of genes from each gene set present in the population of genes detected as under-expressed and over-expressed. *P*-values of the Fisher tests are given for each gene set and each group of genes above each barplot.

Method:

Library construction and RNA-seq
Stranded mRNAseq sequencing has been performed at the Centre National de Recherche en Génomique Humaine (CNRGH, CEA). After complete quality control of RNA on each sample (quantification in duplicate and RNA6000 Nano LabChip analysis on Bioanalyzer 2100 from Agilent), libraries were prepared using the "TruSeq Stranded mRNA Library Prep Kit" from Illumina, with an input of 1 µg and with selection of poly(A) RNAs, following the manufacturer's instructions. Library quality was checked by Bioanalyzer 2100 analysis, and sample libraries were pooled before sequencing to reach the expected sequencing depth. Sequencing has been performed on an Illumina HiSeq 4000 as paired-end 100 bp reads, using Illumina sequencing reagents and pooling 6 samples per lane (corresponding on average to 40 to 50 million sequenced fragments or 80 to 100 million total reads). The quality of the sequences was checked using an in-house CNRGH pipeline before transferring the fasq files to Institut Pasteur.

Bioinformatics analysis
The RNA-Seq reads were mapped to the genome with the STAR aligner v2.5.3a (Dobin et al. 2013) in two-pass mode to the GRCh37 v75genome, STAR was used to output uniquely Mapped read counts at the gene level. The 15,665 genes with at least one count-per-million reads in three samples were selected for further analysis. Differential expression analysis between ASD and Control samples was performed using DESeq2 v1.18.1 (Love et al., 2014). Plots were made in R using the package ggplot2 (Wickham 2009).

Supplementary References:

Darnell, J.C., Jensen, K.B., Jin, P., Brown, V., Warren, S.T. and Darnell, R.B. Fragile X Mental Retardation Protein Targets G Quartet mRNAs Important for Neuronal Function**.** *Cell.* **107**(4):489-99 (2001).

Dobin, A., Davis, C.A., Schlesinger, F., Drenkow, C., Zaleski, C., Jha, S., et al. STAR: ultrafast universal RNA-seq aligner. Bioinformatics, **29,** 15-21 (2013).

Eisenberg, E., and Levanon, E.Y. Human housekeeping genes, revisited. *Trends Genet.* **10**, 569-74 (2013).

Love, M.I., Huber, W., Anders, S. Moderated estimation of fold change and dispersion for RNA-seq data with DESeq2. *Genome Biology*. **15**(12):550 (2014).

Wickham, H. ggplot2: Elegant Graphics for Data Analysis. Springer-Verlag New York, (2009).

Figure S6: Multi-Dimensional Scale Analysis of the genetic distance between individuals of HapMap3 populations and the cohort of control individuals and patients from our study

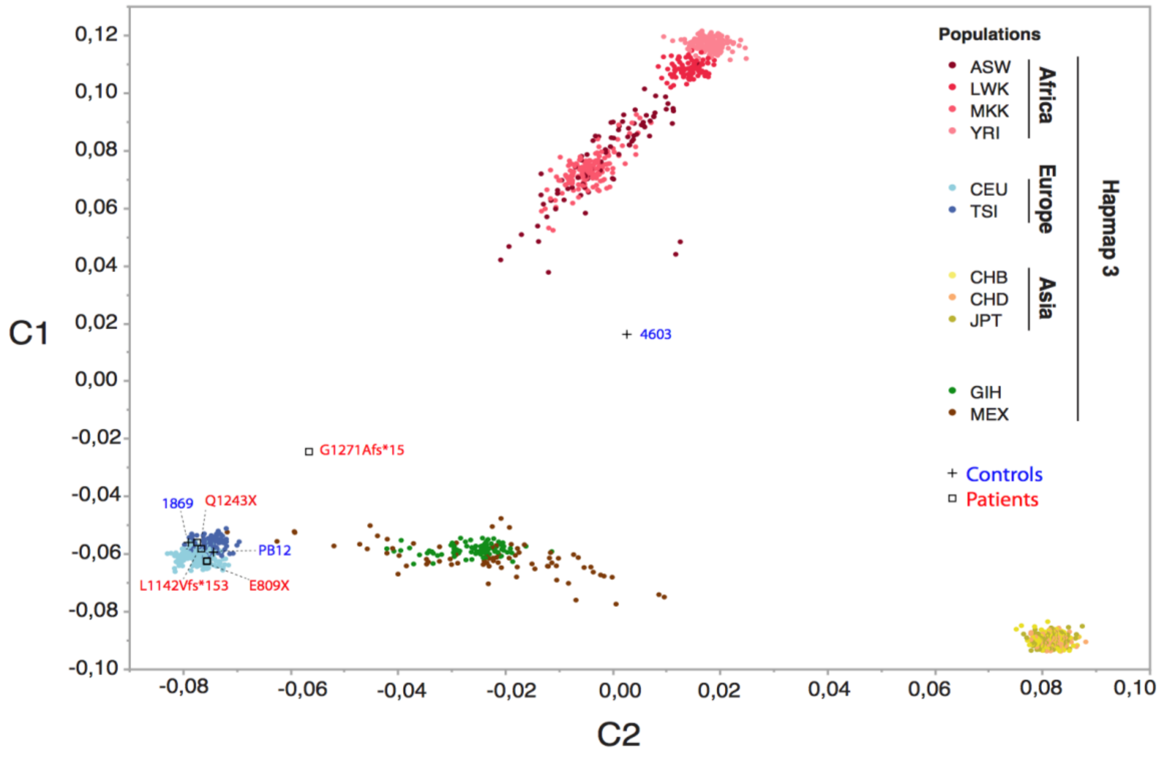

Legend to Figure S6: Each dot represents a single individual. The distance between two dots represents the genetic distances. As described in Materials and Methods, the stratification was performed using PLINK and 17K SNPs overlapping from genotyping data of HapMap3 populations (Illumina Human1M and Affymetrix SNP 6.0) and the cohort of controls and patients (Illumina Infinium Omni1/2.5; 1M/2.5M SNPs) and Illumina Infinium Humancore24 (300K SNPs).

Table S1: Nucleotide sequence of primers used for PCR and Sanger sequencing

Forward		Reverse		Size
21-AF	TGTAAAACGACGGCCAGTTTGTGTCCGGACGGTGGCTTCC	21-AR	GGATAACAATTTCACACAGGACGGAGCGAAGAGGCTGGCGCTGAA	634
21-AF'	TGTAAAACGACGGCCAGTagacatcgcagacgcagact			
21-BF	TGTAAAACGACGGCCAGTGCGCTCCATGATCATCCTGCA	21-BR	GGATAACAATTTCACACAGGACTTTGCCGGTGAGTGGGTGGA	634
21-CF	TGTAAAACGACGGCCAGTGCGGATCTGCCATCCCTACAGC	21-CR	GGATAACAATTTCACACAGGACCACAGCCGCTGACTGCATGG	618
21-DF	TGTAAAACGACGGCCAGTGCTGGCCTCATCGTTGTGCACGCCA	21-DR	GGATAACAATTTCACACAGGACTTGGGAGGCACTGGTGGCTTCTCGA	626
21-EF	TGTAAAACGACGGCCAGTAGGCTGACACACGCAGCTCC	21-ER	GGATAACAATTTCACACAGGACAGGCCAAGCAAGACCGGATTCAG	698

Legend to Supplementary Table S1: Nucleotide sequence of primers used for PCR and Sanger sequencing. Five sets of primers were used to cover the whole sequence of exon 21. Primers were linked to the M13 adaptor sequence (red characters). Three sets of primers (duos B, C and D) were used for genomic DNA and cDNA amplification. Set A and E were not used for cDNA since AF and ER were located in intronic regions. Sequencing was performed using M13 sequences as primers.

Table S2: Commercial antibodies used for immunofluorescence labeling

	HOST	REFERENCE	SUPPLIER	DILUTION
**Primary Antibodies**				
Synapsin	Rabbit	ab1543	Millipore	1/1000
VGlut1	Rabbit	135303	Synaptic System	1/1000
Pan SHANK	Mouse	MABN24	Millipore	1/500
BetaIII tubulin	Chicken	ab9354	Millipore	1/1000
GFAP	Rabbit	ab7260	Abcam	1/1000
MAP2	Mouse	MAB 3418	Millipore	1/200
PSD95	Rabbit	ab18258	Abcam	1/1000
**Secondary Antibodies**				
Anti mouse Alexa 488	Chicken	A21200	Invitrogen	1/200
Anti mouse Alexa 594	Goat	A11005	Invitrogen	1/200
Anti rabbit Alexa 488	Rabbit	A11034	Invitrogen	1/200
Anti chicken Alexa 546	Goat	A11040	Invitrogen	1/200

Table S3: List of coumpounds and Materials used for RT-ddPCR

Primers				
Primers	Forward	Probe	Reverse	Reference
Shank3 Exon 2-3 HEX Home-Made design	GCAAGTTCCTGGATGAGGAG	CCTGCCCTACCTGGAGTTTC	CATAAACTCGCCGCTTGTATC	10031279 (Biorad)
Shank3 Exon 2-3 FAM Home-Made design	CTGGGGGAGGAACCAGTTG	CTGGACCCTGCCAAGAAGT	CACCGAGGCTGCTGAAGAG	10031276 (Biorad)
Shank3 Exon 8-9 HEX Biorad design	NA	NA	NA	10031255 (Biorad) dHsaCPE5058813
Shank3 Exon 8-9 FAM Biorad design	NA	NA	NA	10031252 (Biorad) dHsaCPE5058812
Shank3 Exon 21-22 HEX Home-Made design	CTGGGGGAGGAACCAGTTG	CTGGACCCTGCCAAGAAGT	CACCGAGGCTGCTGAAGAG	10031279 (Biorad)
Shank3 Exon 21-22 FAM Home-Made design	CTGGGGGAGGAACCAGTTG	CTGGACCCTGCCAAGAAGT	CACCGAGGCTGCTGAAGAG	10031276 (Biorad)
GAPDH HEX Biorad design	NA	NA	NA	10031255 (Biorad) dHsaCPE5031597
GAPDH FAM Biorad design	NA	NA	NA	10031252 (Biorad) dHsaCPE5031596

Table S3 (cont.)

Products	
Produts	Reference
ddPCR Supermix for probes no UTP	186-3025 BioRad
DG8TMCartridges for QX100 TM/QX200 TM Droplet Generator	186-4008 Biorad
Droplet Generator DG8 TM Gasket	1863009 Biorad
Droplet Generator DG8 TM Cartridge Holder	1863051 BioRad
Droplet Generator Oil for Probe	186-3005 Biorad
twin.tec PCR Plate 96, semi skirted	951020320 eppendorf
QX200™ Droplet Generator	1864002 BioRad
QX200™ Droplet Reader	1864003 BioRad
QuantaSoft™ Software Regulatory edition	1864011 BioRad
